# Nanotechnology-driven strategies to overcome azole resistance in *Phoma arachidicola*: mechanistic insights, resistance dynamics, and translational barriers

**DOI:** 10.3389/fpls.2026.1772014

**Published:** 2026-02-17

**Authors:** Jinhui Xie, Xue Pei, Chaoqun Zang, Aleksandra O. Utkina, Asim Abbasi

**Affiliations:** 1 Institute of Plant Protection, Liaoning Academy of Agricultural Sciences, Shenyang, China; 2 Institute of Environmental Engineering, RUDN University, Moscow, Russia; 3 Department of Entomology, University of Agriculture, Faisalabad, Pakistan

**Keywords:** ecotoxicologyand regulatory considerations, nanoformulated azole fungicides, nano-fungicides delivery systems, nanotechnology, peanut web blotch, precision agriculture, resistance evolution, sustainable crop protection

## Abstract

Azole fungicides are central to peanut disease management, yet their durability is increasingly jeopardized by the accelerating emergence of azole-resistant fungal populations, including *Phoma arachidicola*, the causal agent of peanut web blotch. Conventional azole formulations exhibit intrinsic physiochemical limitations such as poor aqueous solubility, rapid photothermal degradation, and inconsistent field retention, that drive sub-optimal dosing and intensify resistance selection pressure. Critically, the literature lacks an authoritative synthesis that unifies the mechanistic basis of azole resistance with the design, performance, and translational constraints of nano-enabled azole delivery systems. This review delivers a comprehensive, mechanistically anchored evaluation of nano-assisted azole technologies across polymeric, lipidic, and metallic platforms, interrogating their ability to enhance bioavailability, reinforce cuticular penetration, and disrupt evolutionary pathways that stabilize resistance. In parallel, we rigorously evaluate the persistent regulatory constraints, manufacturing scalability bottlenecks, ecological and regulatory uncertainties, and public acceptance challenges that currently limit the translation deployment of agricultural nanotechnologies. By integrating molecular resistance biology with nanomaterial engineering, and environmental dimensions, this review establishes a decisive framework for designing next-generation azole nanofungicides with unprecedented stability, precision, and resistance-mitigating capacity. The insight articulated herein chart a scientifically grounded roadmap to guide future innovation, regulatory harmonization, and sustainable disease management in peanut agroecosystems.

## 1 Introduction

Peanut (*Arachis hypogaea* L.) stands among the most strategically important oilseed and food legume crops worldwide, underpinning nutritional security and rural livelihoods across tropical and subtropical agroecosystems. As a dual-purpose commodity rich in protein and essential fatty acids, it contributes substantially to both household nourishment and regional economics. Global production is heavily concentrated in China, the United States, India, and Nigeria, which together supply nearly 70% of the world`s peanuts (Janila et al., 2016). Within China, peanut holds exceptional economic and dietary relevance, functioning as a primary source of plant-based protein and edible oil (Jiang et al., 2010), with annual oil output reaching approximately 2.7 million tons and accounting for nearly one-fifth of the nation`s edible oil production (Bodoira et al., 2022). Yet, the crop`s yield trajectory remains chronically constrained by a suite of aggressive fungal disease that compromise canopy health, disrupt physiochemical performance, and collectively result in significant yield and quality losses (Janila et al., 2016).

Among these constraints, peanut web blotch caused by *Phoma arachidicola* (Marasas et al., 1974) stands out as one of the most pervasive yet disproportionately under-investigated foliar diseases threatening global peanut production (Chen et al., 2015). The pathogen is capable of colonizing foliage across all phenological stages triggering progressive chlorotic, necrotic lesions and premature defoliation that typically decline yields by 10–20%, with losses exceeding 50% under favourable epidemic conditions (Liu et al., 2018). Since its first detection in Texas in the 1970s (Pettit et al., 1986), this pathogen has exhibited notable dispersal capacity, becoming entrenched in major peanut-growing regions of China, particularly. Although recent research has advanced understanding of pathogen physiology, epidemiological dynamics, biochemical suppression strategies, and host resistance screening (Liu et al., 2002), these efforts have not translated into consistently effective field management. The scarcity of durable host resistance, coupled with the aggressive disease cycle of *P. arachidicola*, has forced producers to rely heavily on external inputs particularly fungicides highlighting the structural vulnerability of current management strategies.

Consequently, chemical control has remained the dominant approach, with azole fungicides such as propiconazole, tebuconazole, and difenoconazole widely deployed as frontline interventions (Ckromatogr, 1987). Azoles exert profound biochemical effects on plants. Through inhabitation of cytochrome P450-mediated oxidative demethylation, these compounds inadvertently disrupt multiple plant hormonal pathways including abscisic acid, gibberellin, and brassinosteroids biosynthesis (Rademacher, 2015), leading to alterations in growth regulation, metabolic homeostasis, lipid peroxidation, and enzyme activity (Gorshkov et al., 2023). Although experimental studies have documented transient increase in shoots and root elongation, biomass accumulation, and chlorophyll content following azole treatment, mediated by cytokinin-driven modulation of auxin-cytokinin balance and enhanced antioxidant enzyme activity (Maheshwari et al., 2022), these responses reflect stress-induced physiological adjustments rather than directed agronomic improvement. Critically, the widespread and repeated application of azoles, particularly propiconazole and tebuconazole, has exerted intense selective pressure on phytopathogenic populations, including *phoma*species, precipitating the rapid emergence of fungicide resistance and undermining the long-term efficacy and sustainability of disease management (Ishii and Holloman, 2015).

*Phoma arachidicol*a exhibits remarkable genetic and biochemical plasticity, enabling adaptive resistance to azoles through point mutations in the *Cyp51* gene, alterations in sterol biosynthesis pathways and overexpression of efflux transporters (Cools and Fraaije, 2013). These resistance mechanisms significantly compromise the fungicidal activity of azole membrane integrity (Zhang et al., 2021). The consequence is diminished field efficacy, increased production costs, and intensified environmental impacts from repeated chemical applications (Brent and Hollomon, 1995). The escalation of azole resistance in this pathogen underscores the pressing need for a nuanced understanding of molecular, evolutionary, and induced resistance pathways. Despite methodological advancements, traditional resistance monitoring frameworks are constrained by high labour demands, temporal delays, and insufficient throughput, limiting their capacity to provide timely, large-scale insights into the evolution and spread of azole resistance.

In parallel, nanotechnology offers a promising avenue to address these limitations by enabling precise delivery of fungicides, reducing non-target impacts, and potentially overcoming biochemical resistance mechanisms. Nanoformulations, including polymeric, lipid-based, and metal oxide nanoparticles, can enhance azole solubility, improve foliar adhesion, facilitate controlled release, and target pathogen infection sites more effectively (Rai et al., 2009). Encapsulation of azoles within nanocarriers protects active molecules from photodegradation, stabilizes them under variable environmental conditions, and may restore efficacy against resistant fungal populations (Andreadelli et al., 2024). However, translating these innovations into practical field applications is constrained by technical, regulatory, and ecological considerations, including nanoparticle stability, biosafety, and cost-effectiveness.

This review therefore seeks to address these critical knowledge gaps by providing a comprehensive synthesis of current understanding of the disease ecology and molecular mechanisms underpinning azole resistance in *P. arachidicola*. It further critically evaluates the mechanistic principles and functional efficacy of nano-engineered interventions, with particular emphasis on nano-assisted azole delivery and the deployment of multifunctional nanomaterials capable of enhancing pathogen targeting, improving fungicide stability, and mitigating off-target environmental effects. In addition, the review explores emerging nano-biosensing platforms designed for real-time, high-resolution detection of pathogen presence and resistance evolution, offering unprecedented potential for proactive disease surveillance. By integrating these dimensions, the synthesis delineates both the technical and ecological opportunities and constraints of nanotechnology-driven disease management, providing a strategic framework for the development of resistance-smart, environmentally sustainable, and precision-oriented crop protection strategies. Collectively, this analysis not only guides immediate translational applications but also establishes a forward-looking research agenda aimed at overcoming current limitations, enhancing fungicide efficacy, and transforming integrated peanut protection systems into more resilient and adaptive frameworks, with methodologies that can be directly applied to peanuts and systematically investigated in different phytopathogens.

## 2 The *Phoma arachidicola* challenge: disease ecology and the escalating threat of fungicide

### 2.1 Epidemiological landscape and pathogen biology

Peanut web blotch, caused by *Peyronellaea arachidicola* (syn. *Didymella arachidicola* / *Phoma arachidicola*), remains one of the most damaging foliar diseases of peanut in China (Chen et al., 2015; Cui et al., 2016). Since its earliest report from Texas in 1972 (Pettit et al., 1986), the pathogen has spread across major peanut-growing provinces in China, including Henan, Liaoning, Shaanxi, and Shandong during the 1980s to 1990s, highlighting its capacity for rapid geographical expansion (Jun-fan et al., 2013).

The characteristic web-like lesions which are initially needle-shaped gray specks that enlarge into tan, net structured areas develop primarily in the epidermis but may progress to deeper necrosis during severe infection. Under conducive conditions, overwintering conidia germinate and invade epidermal cells, forming a reticulate network that ultimately merges into large blighted regions, leading to defoliation and suppression of pod development (Xie et al., 2023). Yield reductions typically range between 10-20%, but losses may exceed 30% during severe disease outbreaks, imposing substantial constraints on peanut productivity (Ru-jun et al., 2015).

Host range assessment demonstrated that 11 out of 32 tested legume species were susceptible (Pettit et al., 1986), and cultivar-level variation exists, with Virginia and Runner types showing relatively greater tolerance compared to Spanish types. Despite its economic significance, genomic insights into *P. arachidicola* remain limited. The Wb2 strain possesses a ~34.11 Mb genome encoding 37,330 open reading frames (ORFs) enriched in secretory oxidases, peroxidases, and CAZymes implicated in tissue colonization (Zhang et al., 2019). Another strain, YY187, carries a larger genome (47.33 Mb) with a higher G + C content (56.37%), suggesting genomic diversity that may influence virulence and fungicide responses. Because management strategies have been dominated by repeated chemical fungicide applications, concerns over pesticide residues, environmental contamination, resistance emergence, and declining crop quality are intensifying (Culbreath et al., 2021; Luo et al., 2005). Consequently, innovative and sustainable approaches particularly nano-enabled interventions are urgently needed to ensure long-term peanut health and productivity.

### 2.2 Rising fungicide resistance: a persistent evolutionary battle

Sustaining crop productivity depends heavily on chemical fungicides and host resistance, but fungal pathogens have continuously adapted by evolving virulence against resistance genes (Jiang and Tyler, 2012) and by developing decreases sensitivity to fungicides (Avenot and Michailides, 2010). Fungicide resistance refers to the heritable reduction in pathogen sensitivity to a fungicide enabling growth at concentrations inhibitory to sensitive isolates. While terms such as tolerance or insensitivity are sometimes used, the global Fungicide Resistance Action Committee (FRAC) recommends using resistance, now universally applied across microbial control disciplines.

This evolutionary process has led to global proliferation of resistant populations, driving yield losses and contributing to mycotoxin contamination, thereby threatening food security (Chen et al., 2019). Methyl benzimidazole carbamates (MBCs) fungicides were among the first site-specific classes to encounter widespread resistance within just a few years of deployment (Hawkins and Fraaije, 2016).

In peanut pathology, the long-term and unregulated use of triazoles/ Sterol demethylation inhibitors (DMIs) has placed intense selection pressure on *P. arachidicola* populations, accelerating the emergence of azole-resistant isolates (Beg et al., 2025). Key resistance mechanisms include mutations in *Cyp51*, gene amplification, overexpression of the target enzyme, and enhanced drug efflux. Clinical and agricultural experiences further show that widespread azole usage for human and plant pathogens contributes to persistent multi-environment reservoirs of resistance. As a result, azole resistance has now been documented in at least 30 phytopathogenic fungal species across more than 60 countries, underscoring its global scale and critical relevance to crop protection and public health. Current research on optimized derivatives and hybrid azole molecules aims to overcome these complex evolutionary barriers (Teixeira et al., 2022). However, once resistant alleles are established, they are stably inherited and often exhibit cross-resistance within the same mode-of-action group (Yang et al., 2019). DMI resistance is already escalating across several crop systems (Chitolina et al., 2021), signalling similar risks for groundnut production systems.

### 2.3 Understanding azole (DMI) resistance: molecular, evolutionary, and induced pathways

Azole fungicides, classified as demethylation inhibitors (DMIs), have been the backbone of fungal disease control for decades because of their systemic mobility and broad antifungal efficacy. Their primary target, the sterol 14-α-demethylase *Cyp51*, catalyses a critical step in converting lanosterol into ergosterol, a lipid essential for membrane rigidity and cellular homeostasis. When this enzyme is inhibited, ergosterol synthesis collapses and the membrane progressively loses integrity, ultimately killing the fungal cell (Brent and Hollomon, 1995).

**Figure 1:**
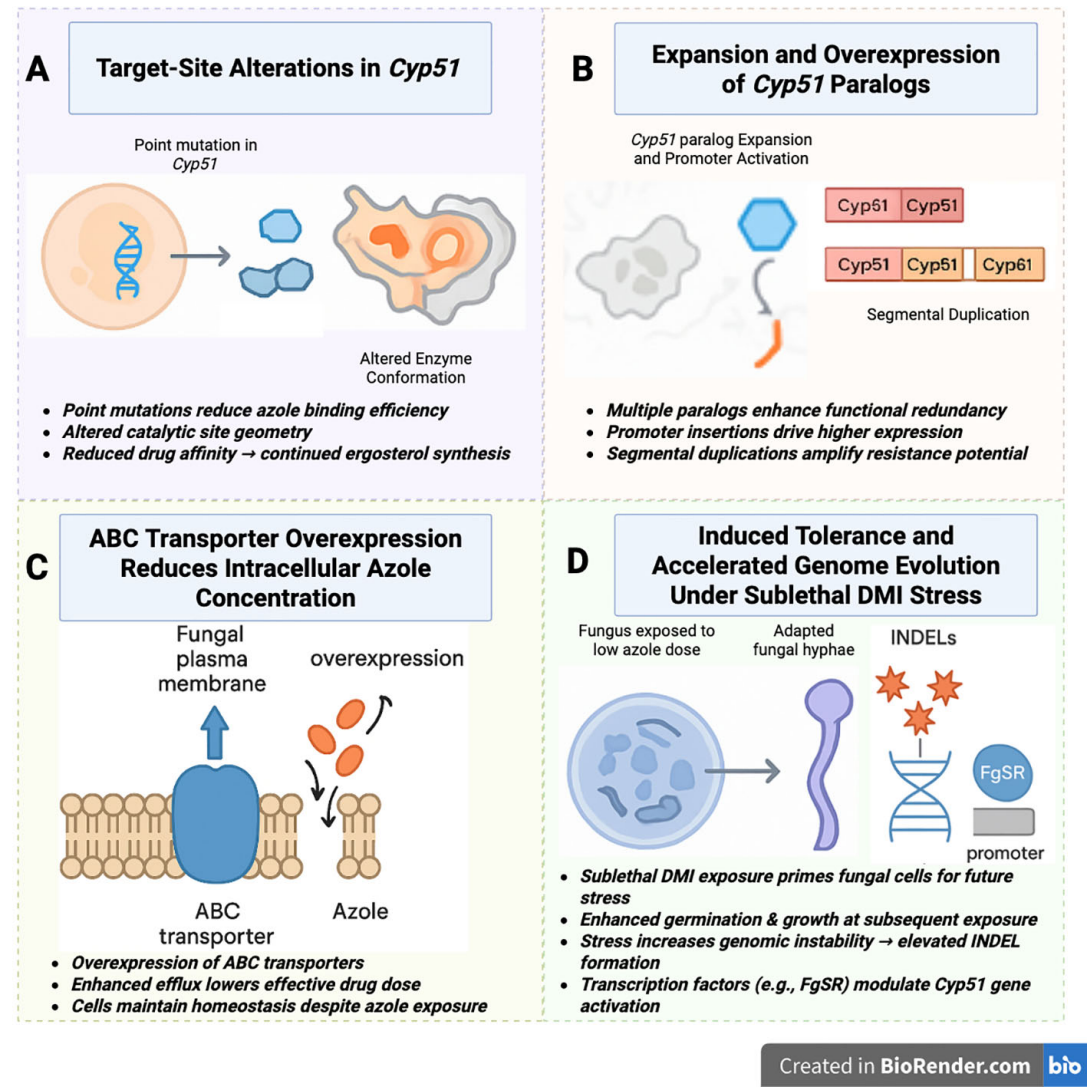
Multilayered mechanisms that drive azole (DMI) resistance in phytopathogenic fungi. Azole fungicides inhibit the sterol 14-α-demethylase *Cyp51*, but fungi deploy diverse adaptive strategies that undermine their efficacy. **(A)** Structural mutations within the *Cyp51* enzyme alter the azole-binding pocket, reducing fungicide affinity and allowing ergosterol biosynthesis to continue. **(B)** Genomic modifications, including the expansion of *Cyp51* paralogs, segmental duplications (e.g., *Cyp51A* duplication), and promoter insertions, enhance enzyme abundance and functional redundancy. **(C)** Efflux pump activation, particularly via ABC transporters, lowers intracellular azole concentrations and diminishes fungicide effectiveness. **(D)** Priming-based tolerance and stress-induced mutagenesis arise when fungi experience sublethal DMI exposure, promoting accelerated INDEL generation, heightened germination during subsequent challenges, and transcription factor–mediated regulation (e.g., FgSR) that collectively facilitate rapid adaptation. Together, these interconnected pathways explain the persistence and escalation of DMI resistance under continuous agricultural use.

Although the inhibitory action of DMI fungicides appears mechanistically simple, phytopathogenic fungi have evolved a highly integrated network of genetic, biochemical, and regulatory adaptation that collectively attenuate or circumvent DMI-imposed selection pressure as showed in **Figure 1**. These adaptive responses include target-site modifications, increased *Cyp51* gene dosage, activation of efflux transporters, and stress-induced tolerance pathways, collectively driving the persistence and escalation of azole resistance. Research across several major plant pathogens including *Fusarium graminearum, Penicillium digitatum*, *Venturia effusa*, and *F. fujikuroi* has revealed that azole resistance does not arise from a single evolutionary route but through diverse and often co-occurring mechanisms (de Chaves et al., 2022).

Resistance to DMIs arises through several mechanisms, including point mutations altering *Cyp51* structure, expansion of *Cyp51* gene copy number, promoter modifications that elevate *Cyp51* transcription, and increase activity of ABC transporter-mediated efflux pumps (**Table 1**). A striking evolutionary dimension to this problem is the variable complexity of the *Cyp51* gene family across fungal taxa. While Saccharomyces cerevisiae and mammals possess only one *Cyp511*gene, majority of filamentous fungi harbor multiple paralogs, sometimes up to four (Celia-Sanchez et al., 2022). *F. graminearum*, for example, possesses three paralogs (*Cyp51*A/B/C), each contributing uniquely to DMI tolerance (Wei et al., 2020). The evolutionary rationale behind these paralogs has long been debated, with hypotheses centered on gene duplication followed by divergent specialization under stress. Compelling support for this view comes from *Rhynchosporium graminicola*, where a large segmental duplication of *Cyp51A* identified in 125 global isolates has been directly linked to emerging DMI resistance in multiple regions (Stalder et al., 2023). Beyond permanent genomic alterations, fungi also exhibit priming-based resistance, a phenomenon increasingly recognized in both clinical and plant pathogenic systems. Priming occurs when exposure to sublethal environmental stressors enhances on organism’s ability to withstand future challenges (Tagkopoulos et al., 2008). In *Aspergillus fumigatus*, subinhibitory concentration of tebuconazole, difenoconazole, or epoxiconazole were shown to increase germination and growth during subsequent DMI exposure, revealing a rapid and reversible form of tolerance (Harish et al., 2022). Even more concerning is evidence that sublethal fungicide stress can induce genomic instability, creating a fertile ground for resistance mutations. Such instability documented in several plant pathogens can accelerate the appearance of adaptive variants (Beckerman et al., 2015). In *Sclerotinia sclerotiorum*, sublethal DMI exposure significantly increased the frequency of INDELs across the genome, offering a mechanistic explanation for the rapid rise of resistance alleles in field populations (Gambhir et al., 2021). Adding further complexity, regulatory networks also modulate the resistance landscape. For instance, the transcription factor FgSR has been shown to bind cis-regulatory elements upstream of *Cyp51*A and *Cyp51*B, orchestrating their expression and enhancing the adaptive capacity of *F. graminearum* under DMI stress (Huang et al., 2019). Taken together, these molecular, evolutionary, and inducible pathways illustrate why azole resistance continues to expand despite decades of stewardship efforts. As agricultural systems remain heavily dependent on DMIs, understanding these mechanisms is critical not only for resistance monitoring but also for designing next-generation, nanotechnology-driven interventions that can mitigate these adaptive advantages and safeguard long-term disease control.

**Table:** Table 1 Mechanisms conferring azole (DMI) resistance across fungal pathogens.

Mechanism	Brief description	Molecular/phenotypic biomarkers	References
Target-site mutations in *Cyp51* (erg11)	Point mutations in 14-α-demethylase that reduce azole binding or access to the active site.	Species-specific substitutions (e.g., *A. fumigatus* L98H, Y121F, T289A; multiple residues in *Zymoseptoria*, *Fusarium*, *B. cinerea*).	(Arnold et al., 2024; Islam et al., 2024)
Promoter alterations (TR-mediated *Cyp51* overexpression)	Tandem repeats or promoter rearrangements elevating *Cyp51*transcription.	TR34, TR46 (*A. fumigatus*); species-specific promoter repeats; elevated *Cyp51* mRNA.	(Chowdhary et al., 2015)
Gene duplication / copy number variation	Increased dosage of *Cyp51* or interacting genes, raising target abundance and decreasing sensitivity.	Elevated gene copy number; presence of *Cyp51*paralogs (A/B/C groups).	(Song et al., 2018)
Trans-acting regulatory mutations	Mutations in transcriptional regulators that upregulate *Cyp51* or sterol-pathway genes.	Increased *Cyp51* and efflux-transporter transcripts; regulatory gene variants.	(Arnold et al., 2024; Islam et al., 2024)
Efflux pump overexpression (ABC/MFS)	Upregulated multidrug transporters reduce intracellular azole levels.	Elevated ABC/MFS genes (e.g., AtrF, CDR1B, MDR1); MDR phenotype.	(Vicentini et al., 2022)
Altered sterol biosynthesis / bypass pathways	Mutations shifting sterol pathway flux or reducing *Cyp51* dependence.	Altered sterol profiles; mutations in sterol-pathway enzymes.	(Wiederhold et al., 2016)
Reduced drug uptake / cell wall remodeling	Cell wall or membrane alterations that limit fungicide penetration.	Modified cell-wall composition or lipid profile.	(Wang et al., 2025)
Combined (multigenic) resistance	Concurrent mechanisms *Cyp51* mutations, overexpression, and efflux producing high-level resistance.	Multiple biomarkers in combination.	(Sen et al., 2024)
Epigenetic / stress-induced tolerance	Reversible, non-mutational responses conferring transient azole tolerance.	Transient gene-expression changes; reversible phenotypes.	(Patra et al., 2022)

## 3 Challenges in monitoring azole fungicide resistance

Effective management of Azole fungicides rely on robust monitoring systems that track resistance frequency, geographic distribution, and underlying mechanisms. Such surveillance informs optimized fungicides programs, including rotation of modes of action, dose adjustments, and risk-based interventions (Hawkins and Fraaije, 2016). The emergence of azole resistant fungal populations has been increasingly reported across diverse agroecosystems, posing a significant threat to crop protection and yield stability. Resistance development is often driven by prolonged exposures to sub-lethal fungicide doses, pathogen genetic plasticity, and selection pressure from repeated applications. Consequently, monitoring systems must not only detect existing resistance alleles but also capture early shifts in population sensitivity and adaptive responses at both field and regional scales. Despite advances in assay technologies, current surveillance approaches remain constrained by their inability to provide continuous, high-resolution data on pathogen population dynamics, limiting the capacity to anticipate resistance evolution and inform timely, evidence-based management strategies.

### 3.1 Cultural and agronomic strategies

Reducing chemical selection pressure through cultural and agronomic strategies is a foundational approach for delaying resistance development. Strategies such as limiting annual fungicide applications, rotating products with distinct modes of action, minimizing eradicant treatments, and integrating non-chemical interventions like resistant cultivars or biocontrol agents have been widely recommended (Brent and Hollomon, 1998a; Corkley et al., 2022). However, the effectiveness of these measures heavily depends on consistent farmer compliance and long-term adoption, which is often variable. Baseline pathogen sensitivity assays, including multi-concentration or discriminatory dose tests, enable early detection of shifts in susceptibility, exemplified by pydiflumetofen resistance screening in *F. graminearum* at 5.0 μg/ml (Shao et al., 2021). Yet, such assays are labour-intensive, require laboratory infrastructure, and are impractical for large-scale or real-time monitoring.

### 3.2 Molecular diagnostic for rapid detection

Molecular diagnostics, including PCR, PCR-RFLP, allele-specific PCR, and real-time PCR allow rapid detection of known resistance mutations at population level (Kunova et al., 2021). Loop-mediated isothermal ampliflication (LAMP) further supports onsite detection within approximately 45 minute and has been successfully applied to pathogens such as *Botrytis cinerea, Podosphaera xanthii*, and *Cercospora beticola* (Shrestha et al., 2020). Despite these advantages, molecular approaches are limited by their reliance on prior knowledge on specific resistance mutations, rendering them ineffective against novel, rare, or polygenic resistance mechanisms. Moreover, they require specialized equipment, trained personnel, and laboratory facilities, which are often inaccessible in many agricultural settings. These methods generally provide only snapshot of pathogen populations, offering limited insight into evolving resistance dynamics or mixed infections in heterogeneous field conditions.

### 3.3 Epigenetic pathways

Epigenetic regulation including DNA methylation and histone modifications has emerged as an additional factor influencing fungicide tolerance, virulence, and stress adaptation (Tang et al., 2021). While clinical inhibitors suggest potential for developing epigenetic fungicides that could bypass conventional resistance mechanisms (Heerboth et al., 2014), practical implementation in crop protection remains largely experimental. The lack of standardized detection methods and field-ready tools further limits the application of epigenetic monitoring for routine resistance surveillance.

These constraints underscore the urgent need for more sensitive, rapid, and integrative detection platforms, providing a strong rationale for exploring nanotechnology-enabled solutions in azole resistance surveillance.

## 4 Nanotechnology for next-generation crop protection

Following the growing challenges in fungicide resistance diagnostics and monitoring, nanotechnology has emerged as one of the most powerful frameworks for next-generation crop protection. The ability of nanoscience to manipulate materials at the atomic and molecular scale has unlocked entirely new modes of agrochemical delivery, plant interaction, and pathogen control, offering breakthroughs that conventional chemistries cannot achieve (Backx, 2020). Nanotechnology deals with structures within the 1–100 nm range, a scale at which matter exhibits quantum-driven physiochemical behaviours distinct from bulk materials (Backx, 2020). These unique properties including high surface-area-to-volume ratios, tunable reactivity, and enhanced bio-interfacing enable the precise engineering of agrochemical formulations with superior efficacy and reduced environmental footprints (El-Bendary and El-Helaly, 2013). Natural nanoparticles, such as oceanic salt aerosols, volcanic dust, and viral particles, demonstrate that nanoscale materials inherently interact with biological systems (Kadar et al., 2014). Synthetic nanoparticles mimic this reactivity, resulting in enhanced capacity to bind, carry, and release biomolecules such as DNA, proteins, RNA, or small molecule pesticides (Albanese et al., 2012).

**Figure 2:**
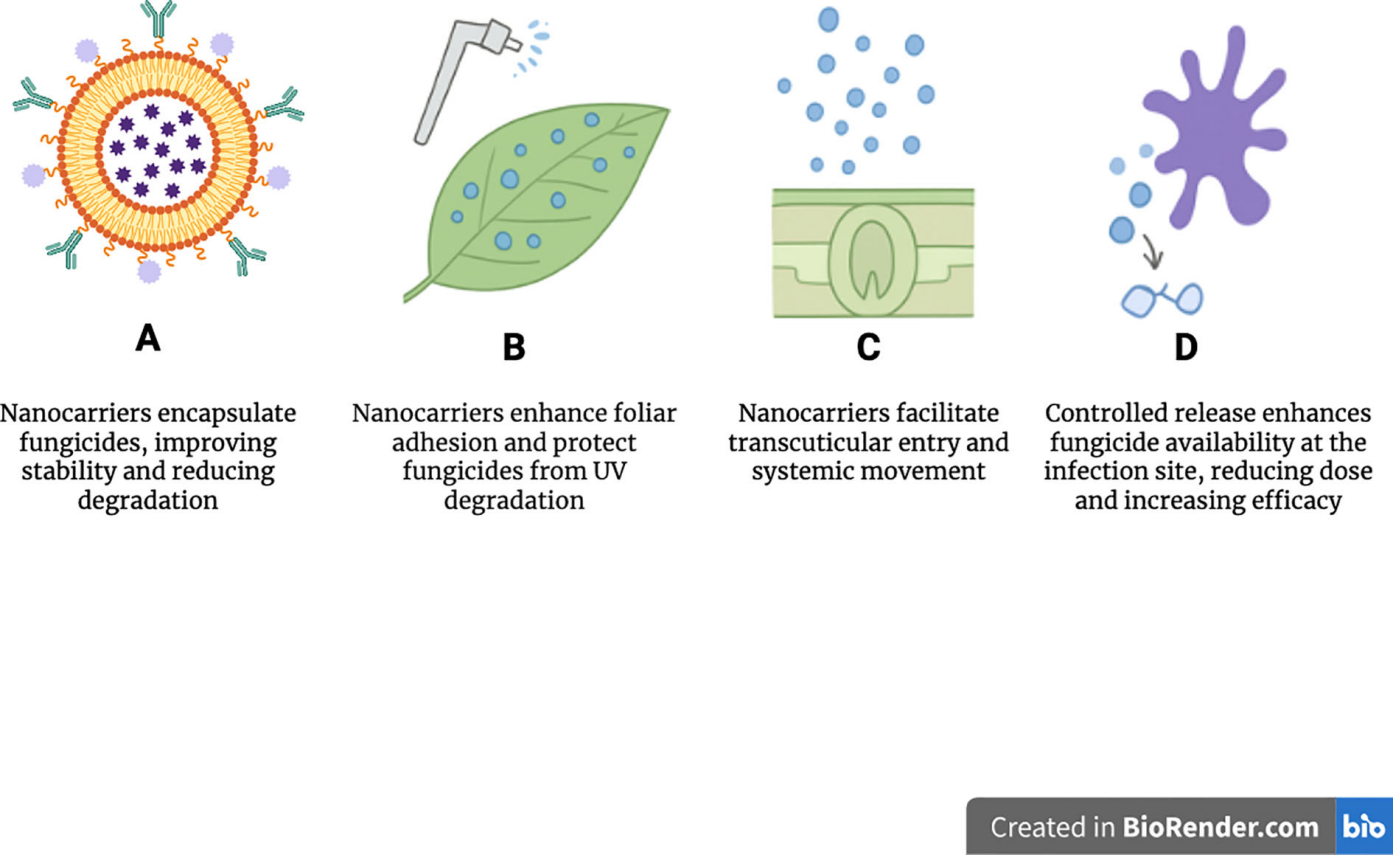
Application of nanocarriers in fungicide delivery. **(A)** Nanocarriers encapsulate fungicides in polymeric, lipid-based, or inorganic shells, improving stability and environmental protection. **(B)** Upon foliar application, nanocarriers enhance leaf surface adhesion and minimize runoff. **(C)** Nanocarriers penetrate plant tissues through stomata and cuticular microchannels and translocate via xylem and phloem. **(D)** Controlled release at infection sites increases local fungicide concentration and improves fungal inhibition.

Nanocarrier-based formulations provide targeted, controlled, and efficient delivery of fungicides, fertilizers, herbicides, and plant growth regulators, significantly reducing off-target effects (Bouhadi et al., 2025). **Figure 2** shows that following foliar application, nanocarriers improve adhesion to leaf surfaces, limit runoff, penetrate plant tissues through stomata and cuticular microchannels, and undergo systemic translocation via the xylem and phloem. The subsequent controlled release at pathogen infection sites increases local fungicide availability and strengthens fungal inhibition. Diverse nanocarriers including biodegradable polymers such as chitosan and PLGA, lipid-based vesicles, silica nanoparticles, and metallic nanostructures are selected to optimize physiochemical stability, compatibility, and release profiles (MaChado et al., 2022). Encapsulation protects active compounds from environmental degradation and enables stimuli-responsive release triggered by pH, temperature, or enzymatic activity. This enhances bioavailability, reduces chemical loss, and improves treatment efficacy across crop systems (Kusumavathi et al., 2025). Nanotechnology thus provides a mechanistically diverse, environmentally responsible platform for next-generation crop protection and fungicide resistance management, aligning with broader sustainability goals by improving resources use efficiency and reducing ecological footprints (Abd-Elsalam, 2024).

### 4.1 Fungal nanoparticle biosynthesis

Nanoparticles can be synthesized using a wide range of physical, chemical, and biological approaches including laser ablation, emulsification, pyrolysis, encapsulation, and dispersion-precipitation (Astruc, 2008). Continuous technological refinement has accelerated the development of novel synthesis routes, making comprehensive categorization a dynamic and evolving process. In parallel, increasing attentions has been directed toward *in vivo* biosynthesis routes employing plants and microorganisms as sustainable alternatives to conventional physiochemical methods (Costa Silva et al., 2017).

Among biological systems, fungi represent particularly efficient platforms for nanoparticle biosynthesis due to their superior protein secretion capacity compared with bacteria and actinomycetes, often resulting in higher nanoparticle yields (Sastry et al., 2003). These distinctive dissimilatory properties allow fungi to mediate rapid, cost-effective, and environmentally benign synthesis of metal nanoparticles. Recognizing this emerging field, Mahendra Rai et al. (2009) introduced the term “myconanotechnology” to encompasses research focused on nanoparticles synthesized using fungal systems. Since then, fungal-mediated nanoparticles synthesized has gained prominence as a powerful green alternative to traditional chemical and physical fabrication methods. Fungi exploit their abundant biomass, extracellular enzymes, and secreted metabolites to reduce metal ions and stabilize nascent nanoparticles, while their inherent bioaccumulation capacity and robust enzymatic machinery further enhance synthesis efficiency (Rami et al., 2024). Fungal nanoparticle biosynthesis can proceed through either intracellular or extracellular pathways; however, extracellular synthesis is generally preferred for large-scale applications due to simplified downstream processing, higher product purity, and improved economic feasibility (Guilger et al., 2017). These advantages closely align with the growing global demand for scalable and sustainable green nanotechnologies.

Microscopic fungi, particularly ascomycetes and imperfect fungi are renowned for producing a vast repertoire of bioactive compounds; nearly 6,400 biologically active molecules have been identified to date (Mahmoud, 2025). Leveraging these metabolic pathways, fungal species such as *Verticillium* sp., *Fusarium oxysporum*, *Penicillium* sp., and *Aspergillus fumigatus* have shown exceptional capability in synthesizing metal and metal sulfide nanoparticles. Compared with bacterial systems, fungi offer several intrinsic advantages, including larger cellular compartments that facilitate substantial metal uptake and a diverse repertoire of extracellular enzymes capable of rapid bioreduction and nanoparticle stabilization. In addition, fungi are easy to culture, highly scalable, and resilient under diverse environmental, collectively supporting their adoption in industrial scale nanomaterial production. These features have led to their recognition as robust and sustainable biological “biofactories” for nanoparticle generation (Eweis et al., 2024).

Despite the greater complexity associated with their eukaryotic organization, particularly in terms of genetic manipulation, recent advances in fungal genomics, transcriptomics, and metabolic engineering are rapidly expanding opportunities to optimize fungal systems for high-efficiency nanomanufacturing. A notable example is the use of *Phoma* species to catalyse specific biochemical reactions leading to the formation of inorganic nanoparticles, representing a rational and emerging biosynthetic strategy. Extracellular mycosynthesis of silver nanoparticles (AgNPs) by filamentous fungi offers the additional advantage of producing large quantities of nanoparticles in a relatively pure form within short timeframes, while simplifying downstream processing (Gade et al., 2013). In this context, the *in vitro* antimicrobial activity of AgNPs synthesized using *Phoma capsulatum, Phoma putaminum* and *Phoma citri* was evaluated against major fungal and bacterial pathogens, including *Aspergillus niger, Candida albicans, Salmonella choleraesuis, Pseudomonas aeruginosa, Staphylococcus aureus* and *Escherichia coli*. All three *Phoma* species produced extracellular, rapid and eco-friendly AgNPs with strong antimicrobial efficacy (Rai et al., 2015). The resulting nanoparticles ranged from 10–80 nm, 5–80 nm, and 5–90 nm, with mean sizes of 31.85 nm, 25.43 nm, and 23.29 nm for *P. capsulatum, P. putaminum*, and *P. citri*, respectively. AgNPs from *P. citri* exhibited the lowest MIC (0.85 µg mL^-^¹) against *S. choleraesuis*, whereas those from *P. capsulatum* showed the highest MIC (10.62 µg mL^-^¹) against *S. choleraesuis, P. aeruginosa*, and clinical *E. coli* isolates. Comparable MIC values (10.62 µg mL^-^¹) were also observed for *P. citri*-derived AgNPs against *P. aeruginosa* and both clinical and standard *E. coli* strains, confirming broad-spectrum antifungal and antibacterial activity (Rai et al., 2015).

Within the broader framework of agricultural nanotechnology, fungal-derived nanoparticles offer distinct advantages. Nanoparticles synthesized through fungal pathways frequently display superior biocompatibility, enhanced structural and chemical stability within soil-plant environments, and reduced risks to non-target and beneficial organisms. Additionally, the incorporation of fungal metabolites during synthesis often confers enhanced antimicrobial and antifungal potency, creating nanoparticles with intrinsic biological activity. These attributes make myogenic nanoparticles particularly attractive for the development of next-generation nano formulated fungicides, nutrient delivery systems, and plant growth enhancers, aligning strongly with the growing demand for eco-friendly, efficient, and sustainable tools in modern crop production.

### 4.2 Nanomaterial classes for managing fungal diseases and resistance

Nanocarriers are broadly classified into four main types: ceramic, carbon-based, organic, and inorganic nanoparticles (**Table 2**). Among these, inorganic nanoparticles consist of both metals and metal oxides, whereas carbon-based nanomaterials include carbon nanotubes, fullerenes, carbon nanofibers, carbon black nanoparticles, and graphene. Additionally, nanocarriers can be categorized according to their structural dimensionality as zero-dimensional, one-dimensional, two-dimensional, or three-dimensional nanostructures (Ijaz et al., 2020). The following subsection summarize the major classes of nanocarriers relevant to fungicide delivery, with emphasis on their structural attributes, mechanisms of action, and applied potential in sustainable crop disease management.

**Table 2:** Categorized overview of nanoparticle-mediated strategies for managing fungal diseases across major crop species, grouped by nanoparticle type and target pathogens.

Target pathogen	Plant disease	Host plant	Mechanism (s) of action	Reported outcomes	Reference
Organic Nanoparticles: Chitosan nanoparticles, Lipid nanoparticles, Polymeric nanoparticles (PLA, PLGA)					
Bimetallic nanoparticles & chitosan nanocomposites	Soil-borne fungi causing seedling damping-off	*Gossypium hirsutum*	Synergistic metal toxicity; ROS-induced cellular damage; chitosan-mediated membrane disruption; inhibition of spore germination	Reduced damping-off incidence; suppressed fungal growth; improved seedling survival and vigor	(Abd-Elsalam et al., 2018)
Carbon Nanostructures (CS)					
CS— *Fusarium graminearum*	Seedling root rot	*Triticum aestivum*	Physical adsorption of spores; disruption of cell walls; possible oxidative damage	Reduced mycelial growth and seedling disease severity in controlled assays.	(Jayaseelan et al., 2013)
Inorganic Nanoparticles: Silver (Ag), Copper (Cu/CuO), Iron oxide (Fe_3_O_4_)					
Ag — *Sclerotium rolfsii*	Collar rot	*Cicer arietinum*	Cell membrane disruption; release of Ag^+^ ions that bind proteins/DNA; ROS generation	Inhibition of fungal growth / reduced disease symptoms; improved seedling health reported.	(Ogunyemi et al., 2019)
Ag — *Fusarium oxysporum*	Fusarium wilt	*Cicer arietinum*	Same as above; interruption of hyphal growth and spore germination	Reduced pathogen colonization and disease severity in treated plants/seed treatments.	(Ghazy et al., 2021)
Ag — *Phytophthora arenaria*	Crown and root rot	*Solanum lycopersicum*	Membrane damage, ionic toxicity (Ag^+^), interference with metabolism	Suppression of pathogen growth and symptom suppression in treated tissue.	(Sarma et al., 2021)
Ag — *Sclerotinia sclerotiorum*	Head rot	*Brassica oleracea* var. *capitata*	Physical interaction with cell wall; silver-ion mediated enzyme inhibition	Lower lesion development and reduced rot incidence on treated heads.	(El-Moslamy et al., 2017)
Ag — *Rhizoctonia solani*	Sheath blight	*Oryza sativa*	Hyphal distortion, leakage of cytoplasmic content, ROS	Decreased mycelial growth and disease symptoms *in vitro*/in planta.	(Guilger-Casagrande et al., 2021)
Ag — *S. sclerotiorum*	Gray mold	*Fragaria ananassa*	Surface adhesion to spores, membrane rupture, ionic toxicity	Reduced sporulation / lesion size in treated fruit/plants.	(Chiranjeevi et al., 2018)
Ag — *Rhizopus stolonifer*	Seed coat discoloration	*Hordeum vulgare*	Spore damage and inhibition of germination	Lower incidence of seed discoloration and improved seed quality post-treatment.	(Elgorban et al., 2016)
Ag — *Phytophthora parasitica*	Black shank	*Citrus limon*	Membrane / enzyme disruption; inhibited zoospore motility	Reduced disease progression on treated plants.	(Abd El-Aziz et al., 2015)
Ag — *F. oxysporum*	Fusarium wilt	*Gossypium hirsutum*	Membrane damage, reduced sporulation	Reduced disease symptoms and pathogen load.	(Ali et al., 2015)
Ag — *F. oxysporum*	Black mold	*Solanum lycopersicum*	Inhibition of spore germination and hyphal extension	Decreased post-harvest mold incidence and lesion spread.	(Sahayaraj et al., 2019)
Au — *Puccinia graminis*	Wheat stem rust	*Triticum aestivum*	(Less biocidal than Ag) likely interferes with spore adhesion/germination and can be used as carrier for antimicrobials	Reported reduction in rust spore germination / potential diagnostic/targeting use; disease suppression in treated samples.	(Ashraf et al., 2020)
Cu — *Aspergillus niger*	Black mold	*Allium cepa*	Release of Cu²^+^ ions, ROS generation, protein/enzyme inactivation	Reduced fungal growth and spoilage on treated bulbs/tissues.	(Kheiri et al., 2016)
Cu — *Poria hypolateritia*	Red root	*Camellia sinensis*	Membrane damage and metabolic inhibition by copper ions	Suppression of root-rot symptoms in treated plants/soil amendments.	(Saad et al., 2018)
Cu — *Penicillium digitatum*	Green rot	*Citrus sinensis*	Copper ion toxicity to spores and hyphae	Lower postharvest decay and fungal load on treated fruit.	(Ponmurugan et al., 2016)
Cu — *Botrytis cinerea*	Gray mold	*Vitis vinifera*	Disruption of cell membrane and fungal respiration by Cu²^+^	Reduced lesion development and sporulation in treated vines/fruit.	(Vanti et al., 2020)
Cu— *A. niger*	Leaf rot	*Eugenia caryophyllata*	Ionic toxicity and membrane disruption	Decreased leaf rot severity after application.	(Hassan et al., 2019)
CuO — *Pythium ultimum*	Pink rot	*Solanum tuberosum*	CuO particle contact + ion release disrupting zoospores	Reduced tuber rot incidence in treated soils or tuber dips.	(Ouda, 2014)
Cu — *Rhizoctonia solani*	Root rot	*Solanum lycopersicum*	Contact killing (particles) and Cu²^+^ mediated enzyme inhibition	Lower disease incidence and improved root health in treated plants.	(Al-Agamy et al., 2023)
MgO — *Aspergillus oryzae*	Brown blotch spot (BBS)	*Oryza sativa*	Alkaline surface reactions; membrane rupture; physical abrasion of spores	Decrease in BBS symptoms and fungal counts on rice tissues.	(Shen et al., 2020)
MnO_2_ — *A. oryzae*	BBS	*O. sativa*	Oxidative stress induction; catalytic ROS generation	Reduction in lesion formation and pathogen viability.	(Shen et al., 2020)
Ceramic Nanoparticles: Silica (SiO_2_), Titanium dioxide (TiO_2_), Zinc oxide (ZnO)					
SiO 2—*Aspergillus flavus*	Ear Rot	*Zea mays*	Cell wall reinforcement; formation of physical barrier; induction of defense-related enzymes; enhanced silicon uptake	Reduced fungal infection; enhanced disease resistance; improved plant vigor and structural strength	(Suriyaprabha et al., 2014)
TiO_2_ — *Bipolaris sorghicola*	Target leaf spot	*Sorghum bicolor*	Photocatalytic ROS generation (under light); cell wall degradation	Reduced lesion size and spore viability under light-exposed conditions.	(Spadola et al., 2020)
TiO_2_ — *B. sorokiniana*	Spot blotch	*Triticum aestivum*	Same photocatalytic & membrane damage effects	Lower spot blotch severity in treated plants/treated seeds.	(Spadola et al., 2020)
ZnO — *Fusarium oxysporum*	Vascular wilt	*Solanum lycopersicum*	ROS induction, membrane damage, interference with spore germination	Reduced disease incidence and improved plant vigor after treatment.	(Zand et al., 2020)
ZnO — *F. culmorum*	Fusarium wilt	*Hordeum vulgare*	Similar ROS / ionic effects; hyphal distortion	Suppressed Fusarium development on treated barley.	(González-Merino et al., 2021)

#### 4.2.1 Biopolymeric-based nanoparticles

Biopolymeric nanoparticles (NPs), derived from either natural or synthetic biocompatible polymers, represent a versatile platform for agrochemical delivery. These nanostructures can be engineered via biological, chemical, and physical routes, offering flexibility in design and functionality. Among emerging strategies, green synthesis using plant extracts, fungi, or bacteria has gained significant attention due to its eco-friendly profile and reduced toxicity (Carbone et al., 2019).

The nanoscale dimensions of biopolymeric NPs confer several agronomically relevant advantages, including enhanced solubility, improved bioavailability, controlled release, and site-specific delivery of active compounds. Further, their high surface-to-volume ratio facilitates stronger interactions with plant tissues and soil matrices, supporting precision agriculture objectives (Mazzinelli et al., 2025). Biopolymeric carriers can be formulated from natural polymers such as chitosan and alginate or synthetic polymers.

Chitosan, a naturally derived, biodegradable, biocompatible, and non-toxic polymer, has emerged as a versatile biopolymeric NP with broad-spectrum antimicrobial, antifungal, antioxidant, antiviral and anti-inflammatory properties (Maluin et al., 2019). In agriculture, chitosan nanoparticles have gained prominence both as potent biostimulants and as antimicrobial agents against a range of phytopathogens. Beyond their direct antifungal activity, they function as efficient nanocarriers for agrochemicals, encapsulating active ingredients through ionic or covalent interactions, or via entrapment within the polymeric chitosan matrix (Maluin et al., 2019). Another study reported the co-encapsulation of citral together with the synthetic fungicide Cyproconazole within solid lipid and chitosan nanoparticle carriers. This dual-loaded nanoformulation exhibited strong antifungal efficacy, with fungal growth suppression observed at a minimum inhibitory concentration of 1.56 µ g mL^−^1, indicating enhanced bioactivity at low doses (Bence et al., 2024). This encapsulation enhances the stability, bioavailability, and controlled release of agrochemicals, aligning with the principles of sustainable and precision agriculture.

Practical applications of chitosan NPS include managing major crop disease such as wilt and *Fusarium* head blight in chickpea and wheat, blast leaf in rice, post-flowering stalk rot in maize, blast disease in finger millet, and leaf spot in maize (Maluin et al., 2019). However, inherent hydrophobicity limits its solubility in water, prompting strategies to combine chitosan with copolymer, organic, or inorganic, matrices to improve dispersibility and functional performance.

#### 4.2.2 Myco-based nanostructures

Fungi have long been recognized for their bioactive potential, and recent advances have harnessed this property to generate mycogenic nanoparticles (NPs) as innovative tools for crop protection. Produced from fungal biomass, these nanoparticles offer a dual advantage: they act as potent antifungal agents while providing a sustainable, environmentally friendly alternative to conventional agrochemicals (Yadav et al., 2023). Integration with other nanotechnologies further enhances their utility, enabling precise pathogen targeting, improved delivery of bioactive molecules, and advanced disease monitoring.

Among fungal-derived NPs, copper oxide nanoparticles (CuONPs) have attracted significant attention. Synthesized via fungal extracts, these CuONPs trigger stress responses in pathogenic fungi, disrupting cellular homeostasis and ultimately causing cell death or irreversible damage. Remarkably, mycogenic CuONPs produced from *P. glomerata* cell free extracts have demonstrated promising efficacy against a broad spectrum of phytopathogens, underscoring their potential as next-generation biofungicides (Ingle et al., 2024). The convergence of fungal biotechnology and nanoscience positions mycogenic nanoparticles as a versatile platform for integrated disease management, their inherent biocompatibility, eco-friendly synthesis, and potent antifungal activity make them ideal candidates for modern agricultural practices, offering a pathway to reduce reliance on chemical pesticides while maintain crop health and productivity. Additionally, myco-based nanoparticles that are derived from nanoparticle by fungal strains have demonstrated great potential to be used in disease management of phytopathogens including phyto-pathogenic fungi (Alghuthaymi et al., 2015).

#### 4.2.3 Solid lipid-based nanodelivery platforms

Solid lipid nanoparticles (SLNs) represent a next-generation lipid-based delivery system in which the lipid core remains solid at room temperature, creating a stable matrix capable of incorporating a wide array of lipophilic agrochemicals. Unlike conventional emulsions, SLNs enable the entrapment of hydrophobic active ingredients without relying on organic solvents, offering a cleaner and more environmentally compatible formulation approach. The rigid nature of the lipid matrix restricts molecular mobility, thus enabling a controlled and prolonged release profile for encapsulated compounds (Borel and Sabliov, 2014). Nonetheless, their utility in agriculture has gained attention, especially for delivering widely used fungicides such as tebuconazole (TBZ) and carbendazim (MBC), both of which remain central to the management of major fungal diseases across crops (Worrall et al., 2018). The enhanced bioactivity of SLN-based formulations has been demonstrated experimentally. In antifungal assays against *A. fumigatus*, the incorporation of natamycin into SLNs significantly boosted its inhibitory performance. The inhibition zone expanded from 18 mm with free natamycin to 26 mm with the SLN formulation, and the minimum inhibitory concentration (MIC) decreased by 2.5-fold in broth dilution experiments, highlighting the potency enhancement achieved through SLN encapsulation (Khames et al., 2019). Overall, SLNs provide a promising nanocarrier platform for delivering lipophilic fungicides with improved efficiency, controlled release, and reduced environmental burden positioning them as a compelling alternative for sustainable fungal disease management in modern agriculture.

#### 4.2.4 Nanoemulsion-based delivery

Nanoemulsions have emerged as an effective strategy for improving the delivery of poorly water-soluble agrochemicals by enhancing their solubility and dissolution behavior. These kinetically stable dispersion typically consists of droplets ranging from 20–200 nm, a size that categorizes them as ultrafine or mini-emulsions (Fernandes et al., 2014). The nanoscale droplet dimensions greatly increase surface area, which in turn improves the dispersion, chemical stability, and bioavailability of hydrophobic active ingredients. The reduced droplet size not only boosts solubilization but also enables deeper penetration of encapsulated molecules into plant tissues, including effective traversal of the cuticular barrier which is considered as an essential feature for maximizing pesticide performance and nutrient uptake (Gupta et al., 2023). Moreover, nanoemulsions can be engineered to exhibit controlled or sustained release profiles, extending the residence time of active agents and reducing the number of field application required. This improved delivery efficiency translates into lower overall chemical dosages, decreasing the potential for off-target effects and limiting environmental contaminations. Consequently, nanoemulsions based formulations contribute to reduced agrochemical runoff and diminished toxicity toward beneficial organisms, promoting a more sustainable approach to crop protection and nutrient management (Kutawa et al., 2021).

#### 4.2.5 Silica-derived nanomaterials for targeted fungicide delivery

Silica-based nanoparticles offer exceptional tunability in morphology, porosity and particle size, making them highly adaptable platforms for agrochemical delivery (Mody et al., 2014). Modern synthesis approaches enable the fabrication of uniform spherical particles with engineered porous frameworks including porous hollow silica nanoparticles (PHSNs) and mesoporous silica nanoparticles (MSNs). These nanoarchitectures act as reservoirs that entrap fungicidal molecules within their inner chambers, effectively minimizing premature volatilization and enabling precision delivery to infection sites. One of the defining advantages of PHSNs is their protective hollow-shell design, which shields encapsulated compounds from photodegradation, particularly under intense UV exposure. Evidence accumulated over the past decade shows that silicon-based interventions enhance plant resilience against a broad spectrum of biotic and abiotic challenges, reinforcing the rationale for incorporating silica nanostructures into next-generation antifungal formulations (Worrall et al., 2018). The functional potential of SiO_2_ nanoparticles has been demonstrated in multiple pathosystems. For example, in carrots, SiO_2_-NPs markedly inhibited *Alternaria dauci* and mitigated disease symptoms. Similarly, biosynthesized silica nanoparticles derived from *A. niger*, characterized by high surface area and compact nano-morphology showed strong activity against early blight in eggplant, positioning them an environmentally favourable alternatives to conventional fungicides (Albalawi et al., 2022).

#### 4.2.6 Antifungal efficacy of metallic nanoparticles against plant pathogens

Metal-based nanoparticles including silver (Ag), copper (Cu), zinc oxide (ZnO), titanium dioxide (TiO_2_), and iron oxide (Fe_3_O_4_) are increasingly recognized as powerful antifungal platforms due to their high reactivity, nanoscale surface properties, and ability to directly disrupt pathogen structures (Kah et al., 2018).

##### 4.2.6.1 Silver nanoparticles

Among metallic nanomaterials, silver nanoparticles (AgNPs) consistently demonstrate the most comprehensive antifungal efficacy, with activity spanning soilborne pathogens, foliar fungi, postharvest molds, and even oomycetes. Their broad-spectrum potential is underscored by the inhabitation of key phytopathogens including *Botrytis cinerea*, *Bipolaris* spp. and *Fusarium* spp., wherein AgNPs effectively suppress mycelial proliferation and spore germination through targeted interactions with the fungal cell wall and disruption of metabolic pathways. Their mode of action also reduces the risk of rapid resistance development, making AgNPs valuable tools in long-term disease management. Synergistic interactions further amplify their utility: co-application with *A. alternata* metabolites or fluconazole enhance growth suppression of *Phoma herbarum, P. glomerata, Fusarium semitectum*, and *Trichoderma* spp., highlighting their potential integration into resistance-management frameworks. Early demonstrations of their cross-species effectiveness such as the effective control of *S. sclerotiorum, Sclerotinia minor*, and *R. solani* reinforced AgNPs as versatile antifungal agents (Min et al., 2009). Subsequent studies expanded this evidence base considerably. Silver PVP/NPs inhibited a wide range of yeasts and molds including *Candida glabrata, C. tropicalis, Aspergillus niger, C. albicans*, and *C. krusei*, confirming their consistent antimicrobial activity across divergent fungal taxa (Bryaskova et al., 2011).

Structural and morphological impacts of AgNPs on pathogens further support their potent antifungal action. Studies reported hyphal deformation and collapse in *Gloeophyllum abietinum, Gloeophyllum trabeum, Chaetomium globosum*, and *Phanerochaete sordida*, as well as dose-and time-dependent inhibition of powdery mildew in pumpkins and cucumbers accompanied by spore lysis and mycelial degeneration. AgNP-assisted photocatalytic systems also show exceptional promise: Ag-doped solid and hollow TiO_2_ nanoparticles, particularly under light irradiation, efficiently suppressed *F. solani* and *Venturia inaequalis* with hollow TiO_2_ particles outperforming pure TiO_2_ due to combined antimicrobial and photocatalytic activity (Boxi et al., 2016). Their efficacy extends to a range of economically important pathogens. At 15 mg L^−1^, AgNPs markedly inhibited *B. cinerea, Rhizoctonia solani, Curvularia lunata, Macrophomina phaseolina, Alternaria alternata*, and *Sclerotinia sclerotiorum*, verifying consistent activity across diverse fungal lifestyles (Krishnaraj et al., 2012). In wheat, AgNPs suppressed conidial germination and blocked infection by *Bipolaris sorokiniana*, preventing the development of spot blotch symptoms observed in untreated leaves (Mishra and Singh, 2015). Comparative assays also revealed that both AgNPs and ZnO NPs and their ionic forms (AgNO_3_ and ZnCl_2_) effectively inhibited *Sclerotinia homoeocarpa* in turfgrasses, indicating that in some contexts ionic release contributes substantially to the antifungal mechanism (Mishra and Singh, 2015).

##### 4.2.6.2 Zinc oxide nanoparticles

Zinc oxide (ZnO) nanoparticles have also gained prominence due to their size-dependent bioactivity. Biogenic ZnO prepared from *Parthenium hysterophorus* extracts suppressed multiple phytopathogens, while ZnO nanomaterials successfully controlled postharvest pathogens such as *Penicillium expansum* and *Botrytis cinerea*, supporting their utility in reducing storage losses. Zinc nanoparticles further reduced *Aspergillus flavus* colonization at 15 mg mL^-^¹, reinforcing their roles in mycotoxin management (Sadeghi et al., 2017).

##### 4.2.6.3 Copper nanoparticles

Among copper-based approaches, copper nanoparticles (CuNPs) show potent inhabitation across a broad fungal spectrum. CuNPs synthesized using the endophyte *Streptomyces capillispiralis* suppressed *Pythium* spp., *Alternaria* spp., *A. niger*, *Fusarium* spp. Similarly, CuNPs significantly inhibited *Phytophthora cinnamomic*, with significantly enhanced suppression when co-applied with copper oxychloride, suggesting that nano-conventional synergism can elevate disease control efficacy (Banik and Pérez-de-Luque, 2017). Parallel findings from field trials indicated that micronutrient formulations containing CuSO_4_ and Na_2_B_4_O_7_ were particularly effective in managing pea rust, providing evidence that copper-based nano-enabled interventions can integrate seamlessly into current field practices (Singh et al., 2013).

## 5 Integrated nano-strategies for sustainable disease management

Balding on the growing understanding of azole resistance challenges in *Phoma arachidicola*, the next step is to explore how nanotechnology reshapes the strategic landscape of peanut disease management. Nano-enabled systems introduces a level of precision, adaptability, and functional integration that conventional fungicide practices cannot achieve. Their value lies not in replacing existing chemistries, but in redefining how these chemistries interact with the pathogen, the plant surface, and environment where resistance is selected.

Within this framework, nanomaterials provide three critical advancements: enhanced azole delivery with controlled deposition and release, multifaceted improvements in fungicide performance through synergistic and physiochemical effects, and rapid nanosensor-based detection tools capable of identifying *P. arachidicola* activity long before lesion appear. Together, these advances represent a forward-looking paradigm in managing azole-resistant early leaf spot, positioning nanotechnology as a cornerstone of next-generation peanut disease control.

### 5.1 Mechanistic insights into nano-assisted azole delivery

The long-term effectiveness of azole fungicides is increasingly constrained by formulation-related inefficiencies that inadvertently accelerate resistance evolution rather than suppress it. Conventional azole formulations suffer from poor aqueous solubility, rapid photodegradation, and heterogeneous deposition on plant surfaces, leading to fluctuating exposure profiles at pathogen infection sites (Gikas et al., 2022). These limitations are particularly consequential for foliar pathogens such as *P. arachidicola*, where repeated cycles of infection and incomplete fungicide coverage favour the persistence and enrichment of azole tolerant subpopulations (Bosch et al., 2014; Sun et al., 2024). In this context, nanotechnology is emerging not merely as formulation upgrade but as a paradigm shift in how azole fungicides interact with both the host plant and the pathogen, redefining precision, persistence, and selectivity in disease control strategies (**Table 3**) (Asmawi et al., 2024).

**Table 3:** Quantitative comparison of azole nanoformulations versus conventional formulations for control of plant pathogenic fungi.

Fungal pathogen	Azole fungicide	Formulation	Applied dose (nano vs conv.)	Quantitative outcome (nano vs conv.)	Quantitative gain	Reference
*Botrytis cinerea*	Tebuconazole	Nano-emulsion vs EC	⅓–½ label dose vs full dose	Field disease severity 18–32% (nano) vs 45–68%(conv.)	59–83% greater disease suppression	(Alghuthaymi et al., 2021)
*Saccharomyces cerevisiae*	Tebuconazole	Mesoporous silica nanoparticles (S1-Teb) vs free tebuconazole	1–10 µg mL^-^¹ (nano) vs same nominal conc. (free)	Significantly higher growth inhibition at lower concentrations for nano-Teb; CFU reduction curves show enhanced efficacy	Higher CFU suppression at equal concentrations and effective control at lower nano-Teb doses (no EC_50_/% values).	(Mas et al., 2014)
Mixed soil/plant fungi	Tebuconazole + carbendazim	PCL nanocapsules / solid lipid nanoparticles vs commercial formulation	0.1–0.7 mg mL^-^¹ (nano) vs equivalent active ingredient dose (conv.)	Encapsulation efficiency >99%; altered release kinetics; plant biomass significantly higher in nano treatments vs commercial at identical doses (fungal inhibition inferred, not directly quantified)	>99% encapsulation with higher plant biomass at equal doses, indicating sustained release (fungal inhibition not quantified)	(Campos et al., 2015)
*Colletotrichum gloeosporioides*	Hexaconazole	Polymeric NPs vs EC	Reduced spray frequency	Disease incidence 20–30%(nano) vs 55–65% (conv.)	~60–70% reduction	(Asmawi et al., 2024)
Multiple phytopathogenic fungi	Tebuconazole	Nano-emulsion vs EC	½–⅓ dose vs full dose	EC_50_ 0.3–0.6 mg L^-^¹(nano) vs 1.2–2.0 mg L^-^¹(conv.)	3–6× lower EC_50_	(Díaz-Blancas et al., 2016)
Multiple plant pathogenic fungi	Propiconazole	Chitosan-based responsive nanofungicide vs EC formulation	Lower applied dose (nano) vs conventional EC	Significantly higher disease control efficacy and reduced oxidative stress at lower doses	Enhanced efficacy and reduced chemical input	(Shi et al., 2026)
Plant pathogenic fungi (laboratory & greenhouse assays)	Difenoconazole	Chitosan nanomicrocapsules vs conventional formulation	Reduced dose (nano) vs label dose (conv.)	Higher antifungal activity and improved disease suppression at reduced dosage (reported numerically *in vitro* and in planta)	Dose reduction with maintained or improved efficacy	(Chang et al., 2024)
Wood-decay fungal system (white- & brown-rot fungi)	Tebuconazole	Oil-in-water nanoemulsion vs conventional treatment	Equivalent tebuconazole loading	Increased fungicide retention in wood; prolonged antifungal protection compared with conventional treatment	Extended persistence and improved local delivery	(Lucia et al., 2021)

EC_50_, effective concentration causing 50% growth inhibition; NPs, nanoparticles; PCL, polycaprolactone; EC, emulsifiable concentrate; CFU, Colony-Forming Unit; Conv, Conventional.

At the biochemical level, azoles target sterol 14α-demethylase (*Cyp51*), disrupting ergosterol biosynthesis and compromising fungal membrane integrity, cellular respiration, and growth (Peyton et al., 2015; Spampinato and Leonardi, 2013). While this mode of action remains highly conserved across filamentous fungi, including *P. arachidicola*, its effectiveness in the field is increasingly undermined by resistance mechanisms such as *Cyp51* overexpression, amino acid substitutions within the azole-binding pocket, and activation of multidrug efflux transports. These mechanisms frequently coexist within individual strains, reflecting strong selection pressure under recurrent azole exposure (Cools and Fraaije, 2013; Whaley et al., 2017). Crucially, such selection pressure is often exacerbated by formulation failure rather than intrinsic fungicide weakness, as sublethal and spatially uneven azole concentrations are potent drivers of adaptive evolution.

Nano-assisted delivery directly addresses this vulnerability by fundamentally altering the physiochemical behaviour of azoles at the plant-pathogen interface. Encapsulation of hydrophobic azole molecules within polymeric, lipid-based, or inorganic nanocarriers shields them from premature degradation while dramatically improving aqueous dispersibility and foliar coverage (Monapathi et al., 2021; Singh et al., 2018). This enhanced dispersion ensures more uniform exposure of fungal propagules to inhibitory concentrations, reducing the probability that resistant individuals survive at spray margins or poorly wetted microhabitats. Empirical evidence from multiple crop systems demonstrates that nano-encapsulated azoles achieve superior antifungal efficacy compared with their conventional counterparts, even at reduced application rates, underscoring the central role of delivery precision in fungicide performance (Ahmad et al., 2023; Campos et al., 2015).

Beyond improved deposition, nanocarriers introduce a second critical dimension: temporal control over fungicide availability. Release kinetics can be precisely tuned by modifying carrier composition, particle size, and matrix cross-linking, enabling sustained delivery that maintains fungicidal concentrations over extended periods. Environment-responsive systems further refine this control, as demonstrated by mesoporous silica nanoparticles that modulate fungicide release depending on moisture or pH conditions (Mas et al., 2014; Wanyika, 2013). Similarly, lignin-based nanocarriers allow programmable release of tebuconazole within plant tissues coupling biodegradability with controlled bioavailability (MaChado et al., 2020). Such sustained-release behaviour is particularly advantageous for managing polycyclic diseases like peanut web blotch, where prolonged protection is required to interrupt successive infection events without repeated fungicide applications.

Nanotechnology also reshapes fungicide-plant interactions in ways that extend beyond delivery efficacy. Reduced contact angles and enhanced adhesion achieved through nanosuspensions increase retention on leaf surfaces, minimizing wash-off losses and environmental contamination while improving uptake at infection-prone sites (Xiao et al., 2022). Surface functionalization further enables targeted interactions with plant tissues, enhancing localization without increasing overall fungicide load. Importantly, emerging multifunctional nanocarriers blur the traditional boundary between crop protection and plant nutrition. Metal-organic frameworks (MOFs)-based systems loaded with hexaconazole have demonstrated not only prolonged suppression of *Ganoderma boninense* but also enhanced seedling growth through gradual nutrient release during carrier degradation, illustrating how nano-enabled fungicides can be integrated into holistic crop health strategies (Farhana et al., 2022).

Despite these advances, the application of azole nano-formulations specifically against *P. arachidicola* remain conspicuously underexplored. Recent genomic and resistance-risk assessments in *P. arachidicola* have identified *Cyp51* and efflux transporters as key molecular determinants of azole sensitivity, providing a robust framework for evaluating nano-enabled interventions (Sun et al., 2024). However, the absence of pathogen-specific nano-azole studies represents both a limitation and an opportunity. Established nano-formulation platforms such as polymeric and solid lipid nanoparticles, gated mesoporous systems, lignin nanocarriers, and MOFs have already demonstrated efficacy against diverse azole-sensitive and azole-tolerant fungi and can be readily adapted for *P. arachidicola* with minimal technological barriers (Campos et al., 2015; MaChado et al., 2020; Mas et al., 2014).

A critical consideration moving forward is that nano-enabled enhancement of azole delivery must be accompanied by rigorous resistance surveillance and environmental risk assessment. Sustained low-level exposure, if poorly calibrated, could inadvertently intensify selection pressure on fungal populations, particularly through efflux-mediated tolerance pathways (Parker et al., 2014). Likewise, the ecological fate of certain nanomaterials especially metallic nanoparticles demands careful evaluation to avoid unintended impacts on non-target microbiota and soil health. Prioritizing biodegradable and biogenic carriers, coupled with molecular monitoring of resistance markers, will therefore be essential for translating nano-azole strategies into durable solutions.

Taken together, nano-assisted azole delivery represents a conceptual shift from simply applying fungicides to engineering their spatial and temporal behaviour within agroecosystems. For peanut web blotch management, this shift offers a powerful opportunity to restore azole efficacy against *P. arachidicola*, slow resistance evolution, and align disease control with broad sustainability goals. The convergence of fungal genomics, advanced materials science, and precision agriculture now sets the stage for next-generation fungicide design where formulation is as critical as the active ingredient itself.

### 5.2 Multifunctional nanomaterials enhancing fungicide performance

**Figure 3:**
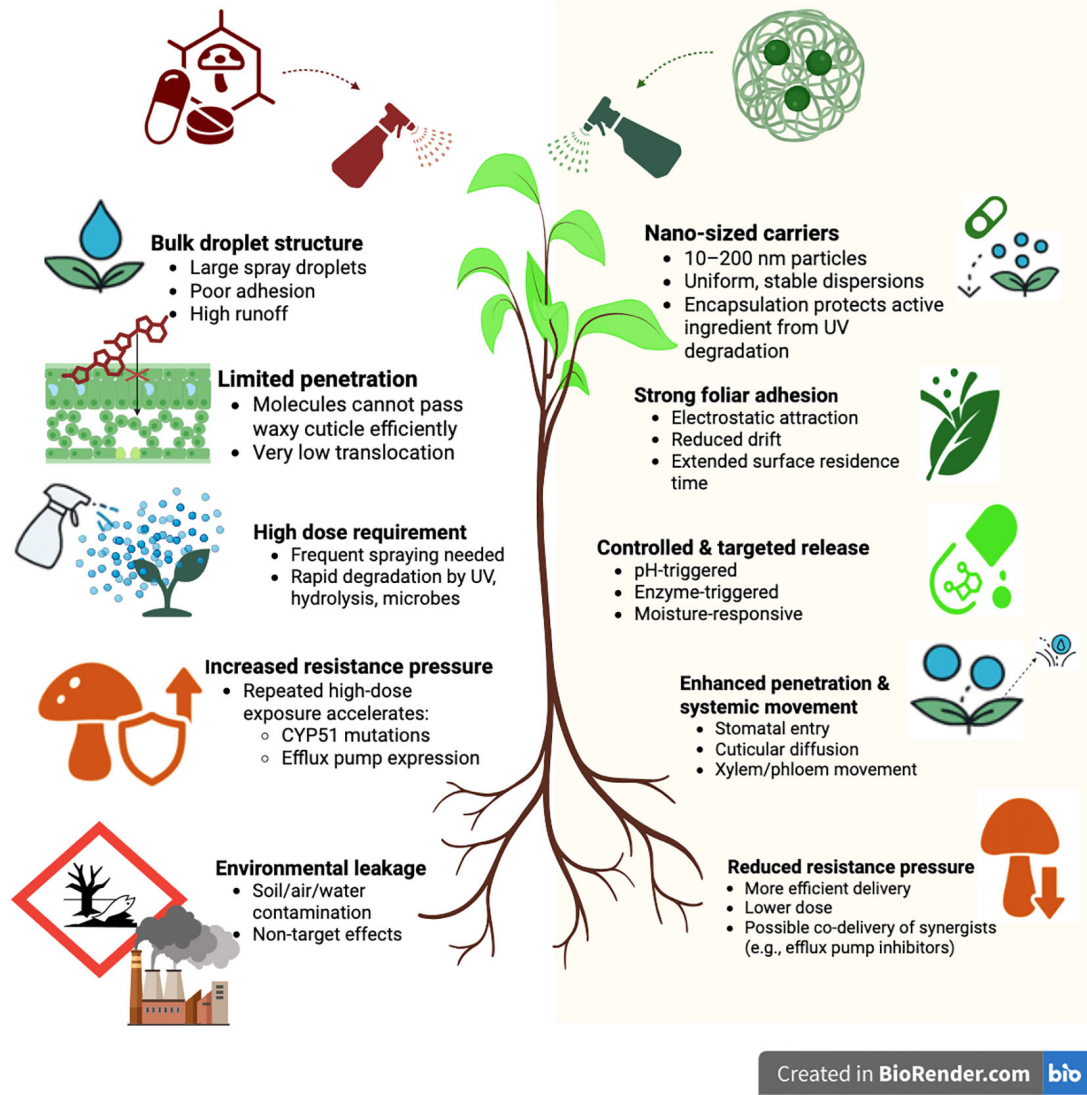
Role of conventional fungicides and nano-formulated fungicides in fungicides performance. Conventional fungicides rely on bulk chemical sprays that show poor leaf adhesion, limited penetration, rapid degradation, and require higher doses, contributing to environmental contamination and stronger resistance selection. In contrast, nano-formulated fungicides utilize engineered nanocarriers that improve foliar retention, enhance trans cuticular and systemic movement, provide controlled release of active ingredients, and achieve higher efficacy at lower doses, ultimately reducing environmental load and resistance evolution.

Conventional fungicides, typically applied as bulk chemical sprays, often exhibited poor foliar adhesion, limited tissue penetration, rapid degradation, and require higher doses, thereby increasing environmental contamination and resistance selection pressure (Brent and Hollomon, 1998b; Popp et al., 2013). By contrast, nano-formulated fungicides leverage engineered nanocarriers to enhance leaf retention, trans-cuticular and systemic transport, and controlled release of active ingredients, resulting in greater efficacy at lower application rates (Dávila Costa and Romero, 2025; Kumar et al., 2025; Sudheep et al., 2025). **Figure 3** illustrates how these nano-enabled systems outperform conventional formulations by integrating physiochemical functionalities such as enhances stability, high surface area, redox activity, and tunable surface chemistry that enable synergistic antifungal actions, broaden activity spectra, and reduce reliance on single-target chemistries. Collectively, these attributes translate into improved persistence, lower environmental footprints, and superior disease suppression, even against recalcitrant or partially resistant pathogen populations.

Functionalization with chemical groups or biomolecules allows nanomaterials to interact more effectively with fungal cells, as demonstrated with multi-walled carbon nanotubes (MWCNTs) functionalized with OH-, COOH-, and NH_2_ groups, which inhibited spore elongation and germination of *Fusarium graminearum* irrespective of nanotube length. Similarly, PEGylated zinc nanoparticles (ZnNPs) exhibited increased activity against multiple *Fusarium* species*, Macrophomina phaseolina, Rhizocotonia solani*, illustrating how surface modifications enhance fungicidal performance (Ammar et al., 2021).

The role of capping agents further underscores the importance of nanoparticle design. AgNPs synthesized via *Trichoderma harzianum* filtrates were significantly more effective when capped with organic residues such as amino acids and proteins, inhibiting mycelial growth and *sclerotia* formation, whereas uncapped NPs failed due to instability and aggregation (Guilger et al., 2017).

Polymeric and inorganic nanomaterials expand this versatility. Chitosan-Cu nanoparticles effectively inhibited mycelial growth and spore germination of *Alternaria solani* and *F. oxysporum*, while Chitosan-Carrageenan nanocomposites loaded with mancozeb achieved up to 83% inhibition of *Alternaria solani* at minimal doses (Kumar et al., 2023). Inorganic nanoparticles such as cobalt and nickel ferrite (CoFe_2_O_4_ and NiFe_2_O_4_) disrupted growth and sporulation of *C. gloeosporioides, F. oxysporum*, and *Dematophora necatrix*, and graphene oxide effectively suppressed *Aspergillus oryzae* and *F. oxysporum* in rice (Sawangphruk et al., 2012).

Encapsulation of hydrophobic fungicides within nanoparticles further enhances their stability, bioavailability, and targeted delivery. Chlorothalonil and tebuconazole, when loaded onto surfactant-free nanoparticles, demonstrated higher uptake and resistance against fungal decay in wood. Similarly, chitosan nanoencapsulation of dazomet and hexaconazole improved uptake and disease control in oil palm against *Ganoderma boninense*, demonstrating the potential of nanaoformulations to optimize both delivery and therapeutic outcomes (Maluin et al., 2019).

Beyond delivery, nanoparticles serve as multifunctional tools for plant protection, combining analytical, antimicrobial, and genetic delivery capabilities. AgNPs exhibit potent activity against *Botrytis cinerea, Rhizoctonia solani, Alternaria alternate*, and *Macrophomina phaseolina*, while copper-based nanocomposites (CuO, Cu2O, and Cu + Cu2O) suppress Phytophthora infestans infections. Zinc nanoparticles enhance both bacterial and fungal resistance, and light-activated TiO2/Zn composites offer targeted suppression of bacterial diseases in plants (Paret et al., 2013). Under greenhouse conditions, ZnO and CuO nanoparticles significantly limited late blight progression in potato plants infected with Phytophthora infestans, achieving substantial reductions in disease severity at low application rates (AlHarethi et al., 2024). Taken together, the evidence underscores how nanomaterials transcend the limitations of conventional fungicide technologies by merging precise delivery, improved persistence, and diverse functional roles, ultimately redefining the framework for sustainable crop protection.

### 5.3 nano-biosensing systems for early pathogen detection

Timely detection remains one of the most decisive factors in preventing fungal epidemics, yet traditional diagnostic tools are slow, labour-intensive, and often reactive rather than preventive. Nano-biosensors address this gap by offering rapid, ultrasensitive detection of fungal spores, toxins, and infection-associated biomarkers directly in the field. Leveraging nanoscale optical, electrochemical, and catalytic properties, these systems can signal pathogen presence within minutes, well before symptoms appear (Ray et al., 2023). By integrating nanosensors into precision agriculture frameworks, farmers gain real-time situational awareness, enabling earlier and more targeted interventions that minimize chemical inputs and avert yield-limiting outbreaks. Through advanced nanosensors, fungal pathogens can now be identified rapidly and precisely, leveraging colorimetric or electrical conductivity changes for near real-time diagnostics (Nainnwal, 2025). This capability overcomes the limitations of conventional methods, which are often slow, labour-intensive, and reactive rather than preventive.

Beyond individual plants, nanosensor-enabled monitoring transforms disease management at the ecosystem scale. Continuous surveillance of soil profile and environmental parameters empowers precision interventions, allowing farmers to anticipate pathogen outbreaks and deploy targeted treatments (Singh, 2023). Such proactive strategies not only contain disease spread but also bolster crop resilience and food security. The economic and environmental advantages are equally compelling. Fungal diseases can reduce yields by 20-40%, threatening both agricultural sustainability and profitability (Ajaz et al., 2024). Nanosensors facilitate rapid agricultural detection and minimize dependency on broad-spectrum fungicides, reducing chemical usage, environmental contamination, and potential human health risks (Ajaz et al., 2024; Sundararajan et al., 2024). Together, these technologies establish a new paradigm for high-precision, sustainable disease management, combing early detection with ecological responsibility.

## 6 Barriers and opportunities in nanotechnology-driven peanut disease management

### 6.1 Scaling biogenic nanomaterials: technical and production constraints

Despite rapid progress in biogenic nanoparticle synthesis, the translation of fungal nanotechnology into field-ready crop protection tools remains constrained by fundamental production and material performance limitations. The agricultural sector demands industrial-scale quantities of highly uniform, bioactive nanomaterials, yet current mycogenic platforms often struggle to deliver consistent particle morphology, monodispersity, and chemical functionalization at economically viable rates. As (Kumar et al., 2023) emphasize, the long-term integration of nanotechnology into plant disease management is inseparable from high-throughput, cost-efficient manufacturing pipelines capable of meeting the volume and quality benchmarks required for commercial fungicide delivery systems.

Beyond manufacturing capacity, the environmental durability of fungal-derived nanomaterials represents a second critical barrier. Nanoparticles deployed in crop fields must withstand an exceptionally dynamic set of abiotic pressures including ultraviolet radiation, temperature oscillations, humidity shifts, phyllosphere hydrodynamics, and soil physiochemical variability. Under such fluctuating conditions, nanoscale systems often experience aggregation, premature release of active ingredients, altered surface chemistry, or loss of functional bioactivity, ultimately compromising field-level performance. Underscores the urgency of developing robust stabilization chemistries, whereas (Mohanty et al., 2024) warn that insufficient stability can undermine the controlled release kinetics essential for maintain sustained antifungal pressure.

These challenges highlight the broader need for next-generation formulation science tailored specifically for agricultural ecosystems. Promising avenues include cross-linked biopolymer matrices that resist environmental degradation, nano-bio hybrid structures that leverage fungal metabolites for enhanced stability, and stimuli-responsive coating engineered to release azoles only under pathogen-inductive cues. Such innovations will be pivotal for ensuring that mycogenic nanoparticles and azole nanocarriers retain their structural and functional integrity from the moment of synthesis through field deployment.

### 6.2 Regulatory frameworks and governance of agricultural nanotechnology: a global perspective

The rapid advancement of nanotechnology research in agriculture has outpaced the development of regulatory systems capable of addressing its unique physiochemical and ecological behaviours. Across jurisdictions, nanomaterials are largely governed through adaptations of existing chemical, pharmaceutical, and food safety legislation rather than dedicated nano-specific frameworks. This has resulted in heterogenous regulatory approaches, varying levels of precaution, and persistent uncertainty for developers and end-users. While some regions emphasize precaution and lifecycle safety, other prioritize regulatory flexibility and innovation, collectively shaping the global governance landscape for fungal nanotechnology and nanofungicides.

#### 6.2.1 European Union

The European Union has adopted one of the most structured and precaution-oriented approaches to nanosafety, centered on the safe-by-design concept, which integrates safety considerations across the entire life cycle of nanomaterials (Gottardo et al., 2017). Regulatory oversight is primarily coordinated through the REACH Regulation EC 1907/2006 (European Commission, 2006), with nano-specific information requirements introduced in 2018 (European Chemicals Agency, 2020; Allan et al., 2021). These requirements apply alongside other sectoral legislation covering food, feed, biocides, occupational safety, and cosmetics. The European Chemicals Agency (ECHA) evaluates nanoforms within chemical registration dossiers and hosts the EU Observatory for Nanomaterials, which aims to improve transparency and public access to information (Sumrein, 2019). Regulatory development is further embedded within broader sustainability initiative, including the European Green Deal and evolving Chemicals Strategy for Sustainability, which increasingly intersect with nanomaterial governance through actions on plastics, microplastics, and circular economy policies (European Commission, 2018, European Commission, 2019, European Commission, 2020; European Union, 2019; United Nations, 2015).

#### 6.2.2 United States of America

The United States follows a regulatory science-driven, case-by-case approach to nanotechnology oversight. The U. S. Food and Drug Administration (FDA) defines regulatory science as the development of tools and standards to evaluate safety, efficacy, and performance, and assumes that existing legislative frameworks are sufficient to regulate nanomaterials (Goering, 2019). Rather than introducing nano-specific laws. The FDA relies on horizon scanning, internal review processes, and guidance development for industry (US Food & Drug Administration (FDA), 2020). Supporting infrastructure includes nanotechnology core facilities and the Collaborative Opportunities for Research Excellence in Science (CORES) programme, which advance method development and interdisciplinary research. Interagency coordination occurs through the National Nanotechnology Initiative (NNI), addressing exposure assessment, environmental and human health, risk management, and informatics (National Nanotechnology Initiative, 2020; Friedersdorf, 2019). International regulatory alignment is strengthened through EU-US collaboration, including mutual recognition agreements and shared inspection frameworks (Nalubola, 2019). Despite these efforts, the number of approved nano-enabled products remains limited, with many applications still progressing through clinical or pre-market evaluation stages (Tyner, 2019).

#### 6.2.3 Canada

Canada applies a risk based-regulatory approach aligned with Organization for Economic Co-operation and Development (OECD) guidance, coordinated through the Chemical Management Plan (CMP). Multiple agencies contribute to lifecycle-based assessments, market surveillance, and exposure evaluation of nanomaterials under existing chemical, food, feed, and pesticide legislation (Health Canada, 2020). Canadian regulatory practice closely aligns with the United States through the Canada-US Regulatory Cooperation Council, enabling shared classification schemes and joint risk assessment activities (OECD, 2020, OECD, 2020). Canada has been at the forefront of nanomedicine development, particularly for non-viral nanoparticles-based drug and gene delivery systems. However, regulatory challenges persist, especially for individualized or small-batch nano-enabled products (Cullis, 2019). Canadian agencies also maintain close collaboration with European regulators through OECD-led initiatives.

#### 6.2.4 China

In China, the regulation of nanotechnology-based agricultural products is distributed across multiple governmental authorities, including the Ministry of Agriculture and Rural Affairs (MARA), the State Administration for Market Regulation (SAMR), the National Health Commission (NHC), and the Ministry of Ecology and Environment (MEE) (Kumari et al., 2023). Early regulatory oversight emerged through the Regulations on the Safety Assessment of Agricultural Genetically Modified Organisms (MARA Order No. 7, 2001), which established approval procedures for advanced agricultural technologies, including applications integrating nanotechnology. Food related applications are governed by the Safety Requirements for Food and Food Additive Containing Nanomaterials (NHC No. 13, 2011), which define evaluation principles for nano-enabled inputs entering the food chain. To address agriculture-specific nonapplications, MARA issued the Technical Guidelines for Safety Assessment of Nano-Scale Agricultural Products (MARA No. 198, 2014), providing structures criteria for assessing production, processing, and field use (Kumari et al., 2023). More recently, regulatory refinement has continued through the Administrative Measures for Safety Evaluation of New Varieties of Agricultural Genetically Modified Organisms (SAMR Order No. 8, 2020) and the Measures for the Administration of Environmental Safety Assessment of Agricultural Genetically Modified Organisms (MEE Order No. 12, 2021), which explicitly extend safety evaluation to environmental exposure pathways relevant to nano-enabled agricultural products (http://en.nim.ac.cn/; http://en.nim.ac.cn/division/overview/924) (Kumari et al., 2023).

#### 6.2.5 Regulatory gaps and governance needs

Despite region-specific advances, global governance of agricultural nanotechnology remains fragmented. Conventional regulatory paradigms fail to capture nano-particle-specific properties such as quantum-level reactivity, altered bioavailability, and nanoscale mobility in soil-plant systems (Servin et al., 2015). This structural mismatch leads to systematic underestimation of both risks and benefits, undermining the scientific credibility of regulatory decisions and constraining the safe translation of laboratory innovations into field-ready applications.

A central limitation is the persistent absence of nanomaterial-tailored risk assessment frameworks. As emphasized by (Abd-Elsalam, 2024), the lack of standardized approaches for evaluating nanoparticle aggregation dynamics, trophic transfer, environmental persistence, and transformation across agricultural matrices represents one of the most significant barriers to commercialization. Without such tools, regulatory agencies are unable to establish robust exposure thresholds, residue limits, or evidence-based application guidelines. This uncertainty disproportionately affects nano-enabled fungicides and azole nanoformulations, where delivery efficiency, controlled release, and resistance management benefits cannot be adequately recognized within existing regulatory dossiers.

Regulatory incoherence across national jurisdictions further compounds these challenges. Divergent definitions of nanomaterials, inconsistent data requirements, and non-aligned evaluation criteria lead to duplicated testing, extended approval timelines, and limited cross-border acceptance of nano-enabled products (Prasad et al., 2017). For agricultural nanotechnologies intended for deployment across diverse agroecological zones, such as peanut-based production systems this lack of harmonization not only restricts farmer access to safer and more effective disease management tools but also disincentivizes private-sector investment and large-scale field validation.

Looking forward, regulatory evolution must shift from substance-centric hazard screening toward function- and exposure-driven governance models. Science-driven frameworks, as articulated by (Kookana et al., 2014), should integrate clear and operational definitions of agricultural nanomaterials, validated nano-specific test protocols, and full life-cycle risk assessment spanning synthesis, formulation, application, environmental transformation, and end-of-life fate. Importantly, future governance structures must move beyond single-product evaluation and explicitly address system-level interactions within agroecosystems.

In this context, next-generation risk models must incorporate long-term, cumulative, synergistic, and antagonistic interactions between nanoformulations, conventional agrochemicals, soil properties, climatic variables, and biological communities (Balusamy et al., 2023). The integration of predictive modelling, tiered testing strategies, and adaptive regulatory pathways would enable regulators to account for dynamic environmental behaviour while reducing unnecessary testing burdens. Such approaches would also facilitate iterative refinement of regulatory thresholds as new scientific evidence emerges.

Ultimately, effective governance should be seen not merely as regulatory compliance but as a catalyst for innovation. Transparent, adaptive, and internationally harmonized frameworks are critical for building public trust, enabling responsible industrial investment, and accelerating the safe deployment of fungal nanotechnology and azole nanoformulations. aligning regulatory science with technological progress will be essential to realizing nanotechnology`s full potential as a cornerstone of sustainable, resistance-resilient disease management in peanut and other cropping systems.

### 6.3 Environmental fate, persistence, and ecotoxicological implications

A critical frontier for nano-enabled fungicide resistance management lies in examining how biogenic and azole-loaded nanocarriers behave, degrade, and influence ecological health. Nanoparticles introduced into soil or sprayed on foliage undergo continuous physiochemical transitions (aggregation, dissolution, corona formation, and redox reactions) that redefine their mobility and biological interactions. These transformations determine nanoparticle retention, leaching potential, plant uptake pathways, and their influence on microbial guilds that regulate nutrient cycling (Elmeanawy et al., 2022) emphasize that only integrated models bridging nanotechnology, soil biogeochemistry, microbial ecology, and plant physiology can reliably predict such multi-layered interactions across time.

A parallel challenge is persistence and degradation behaviour, which varies widely with carrier composition and environmental context. Surfactant-based systems show contrasting mineralization patterns; lecithin degrading rapidly while synthetic surfactants like polysorbate 80 degrade slowly, raising concerns about long-term soil accumulation (Maluin et al., 2019). Polymeric nanocarriers likewise differ: PLA persists longer than PLGA due to slower hydrolysis (Elsawy et al., 2017), while elevated temperatures or alkaline pH accelerate breakdown (Sharma, 2019). Such variability complicates predictions about environmental residence times and long-term ecological consequences, particularly under repeated application cycles typical of disease management programs.

These scientific uncertainties directly intersect with public perception, which can influence regulatory decision and adoption trajectories. Without transparent, longitudinal data demonstrating safe degradation pathways and low ecotoxicological risk, public acceptance of nano-enabled agricultural interventions may remain limited. Thus, environmental fate studies are central to the ethical and socially responsible deployment of nanotechnology in modern crop protection. Together, these complexities underscore the necessity for comprehensive environmental life-cycle assessments, improved material design for controlled degradation, and open science communication to foster public trust. Advancing these domains will determine whether they can achieve broad, sustainable integration into agricultural disease management.

### 6.4 Formulation stability in azole-based nanofungicides

Although azole-loaded nanoparticles offer a strategic advantage over conventional fungicide formulation especially for mitigating resistance evolution, the stability of these nanosystems remains a key determinant of field performance. Lipid-based nanocarriers, nanoemulsions, and vesicular systems are particularly sensitive to variations in pH, ionic strength, and temperature, all of which are common stressors in real agricultural environments. When these systems destabilize, premature drug release and droplet coalescence undermine targeted delivery, compromising disease control efficacy (Hassanzadeh et al., 2022).

A major pillar of stability engineering lies in precise optimization of the Hydrophilic-Lipophilic Balance (HLB) of excipients. It was demonstrated that excipients with carefully tuned HLB values support more durable emulsions, improved structural cohesion, and controlled azole release profiles (Asmawi et al., 2019). Mixed surfactant systems such as mixtures of Tween 80 and Tween 60 enhance interfacial packing density and molecular interactions, resulting in superior droplet stabilization (Chang and McClements, 2014) further showed that formulations incorporating medium-chain triglycerides or orange oil yield exceptionally stable nanosystems when combined with high-HLB surfactants. Similarly, revealed that tuning the Tween 80: Span 20 ratio to achieve an HLB of 3:1 produced the smallest and most stable nanocarriers, underscoring the centrality of surfactant engineering in advanced azole delivery platforms. These findings collectively demonstrate that excipient selection, interfacial design, and molecular-level tuning are not minor formulation decisions, instead they are strategic levers that define whether azole Nanofungicides can maintain integrity across agricultural environments.

## 7 Conclusion and Future Prospects

Conventional strategies for managing peanut fungal diseases, particularly web blotch, caused by *Phoma arachidicola*, are increasingly compromised by rapid azole resistance, narrow-spectrum efficacy, environmental contamination, and escalating production costs. Nanotechnology provides a paradigm shift by enabling precision-targeted azole delivery, multifunctional nanomaterials, and nanoscale biosensing systems that enhance solubility, stability, foliar adhesion, and pathogen-specific uptake, while simultaneously reducing off-target environmental impacts. Nevertheless, the intrinsic risk of resistance evolution necessitates integration with cultural practices, fungicide rotation, biological control agents, and deployment of resistant cultivars to sustain long-term efficacy.

Future research must focus on the design of smart, stimuli-responsive nanocarriers capable of on-demand fungicide release triggered by pH, enzymatic activity, or environmental cues, and on the development of nano-biohybrid systems that synergistically combine nanoparticles with beneficial microbes to amplify antifungal activity, fortify plant immunity, and improve soil health. Integration of artificial intelligence, machine learning, and high-throughput screening can accelerate rational nanocarrier design, optimize physicochemical properties, and predict environmental behaviour, thereby improving efficacy and biosafety. Comprehensive field-level investigations are essential to elucidate nanoparticle persistence, degradation dynamics, impacts on non-target organisms, and interactions with the soil microbiome, while harmonized regulatory frameworks, residue standards, and farmer-centered adoption strategies will ensure safe, equitable, and globally compatible deployment. By merging mechanistic insights into pathogen resistance with nanotechnology-enabled precision delivery, diagnostics, and multifunctional control, this approach can redefine peanut disease management, offering a replicable model for other crops and pathogens and enabling resilient, adaptive, and environmentally sustainable agricultural production systems.
